# Nonlinear and Linear Measures in the Differentiation of Postural Control in Patients after Total Hip or Knee Replacement and Healthy Controls

**DOI:** 10.3390/diagnostics12071595

**Published:** 2022-06-30

**Authors:** Anna Hadamus, Michalina Błażkiewicz, Aleksandra J. Kowalska, Kamil T. Wydra, Marta Grabowicz, Małgorzata Łukowicz, Dariusz Białoszewski, Wojciech Marczyński

**Affiliations:** 1 Department of Rehabilitation, Faculty of Medical Sciences, Medical University of Warsaw, 02-091 Warsaw, Poland; marta.grabowicz@wum.edu.pl (M.G.); dariusz.bialoszewski@wum.edu.pl (D.B.); 2 Faculty of Rehabilitation, The Józef Piłsudski University of Physical Education in Warsaw, 00-809 Warsaw, Poland; michalina.blazkiewicz@awf.edu.pl; 3 Professor Adam Gruca Independent Public Teaching Hospital in Otwock, Rehabilitation Clinic, 05-400 Otwock, Poland; aleksandra.macheta@wp.pl (A.J.K.); kamil.wydra@interia.eu (K.T.W.); mlukowicz@cmkp.edu.pl (M.Ł.); 4 Medical Centre for Postgraduate Education, 01-813 Warsaw, Poland; wmarczynski@interia.pl

**Keywords:** hip arthroplasty, knee arthroplasty, older adults, postural control, body balance, osteoarthritis, sample entropy, fractal dimension, Lyapunov exponent

## Abstract

Primary osteoarthritis treatments such as a total hip (THR) or knee (TKR) replacement lead to postural control changes reinforced by age. Balance tests such as standing with eyes open (EO) or closed (EC) give a possibility to calculate both linear and nonlinear indicators. This study aimed to find the group of linear and/or nonlinear measures that can differentiate healthy people and patients with TKR or THR from each other. This study enrolled 49 THR patients, 53 TKR patients, and 16 healthy controls. The center of pressure (CoP) path length, sample entropy (SampEn), fractal dimension (FD), and the largest Lyapunov exponent (LyE) were calculated separately for AP and ML directions from standing with EO/EC. Cluster analysis did not result in correct allocation to the groups according to all variables. The discriminant model included LyE (ML-EO, ML-EC, AP-EC), FD (AP-EO, ML-EC, AP-EC), CoP-path AP-EC, and SampEn AP-EC. Regression analysis showed that all nonlinear variables depend on the group. The CoP path length is different only in THR patients. It was concluded that standing with EC is a better way to assess the amount of regularity of CoP movement and attention paid to maintain balance. Nonlinear measures better differentiate TKR and THR patients from healthy controls.

## 1. Introduction

Osteoarthritis (OA) is a multifactorial disease leading to cartilage degeneration and damage to the surrounding tissues: joint capsule, ligaments, subchondral bone, periarticular muscles, and nerve endings. As a chronic disease, it leads to biomechanical changes in the affected joint as well as burdensome symptoms such as pain, stiffness, swelling, and loss of function. In advanced stages, OA can lead to severe physical impairment [1,2]. Osteoarthritis frequency increases with age and most often involves big joints in the lower limb: hip or knee joint being the dominant source of disability, affecting approximately 776 million people globally [3].

Patients in advanced stages of OA, with persistent pain, loss of function, and advanced radiographic changes are qualified for joint arthroplasty, which is an effective (also cost-effective) procedure, giving much better results than physical therapy programs [4]. Current concepts do not recommend arthroscopic debridement for treating OA. Additionally, arthroscopic partial meniscectomy has a limited role in patients with symptomatic meniscal tear coexisting with knee OA [4]. The number of total hips (THR) and knee replacement (TKR) surgeries has increased rapidly over the last decades [2,3,5]. The incidence rate of TKR in the US population was 272 per 100,000 citizens in 2002, and 429 in 2012, and it is expected to increase by 143% by 2050 [6]. More than 300,000 primary total hip replacements and over 700,000 primary total knee replacements are performed annually in the US, of which more than 90% are due to OA [4]. Symptoms of OA, as well as invasive procedures such as THR or TKR, affect joint function. Due to damaged and/or cut nerve endings and roots, long-lasting pain, and damage to the joints and surrounding tissues, proprioception and motor control in this area are often severely affected. This leads also to postural control changes, which are reinforced by age by impairing the capability of the central nervous system to process signals from somatosensory, visual, and vestibular networks [7]. After both types of surgery—THR, and TKR—leg length discrepancy is often observed [8,9]. Anatomical discrepancies are corrected during the arthroplasty, but functional changes, including movement habits, remain in these groups of patients unchanged. Leg length discrepancy of 2 cm or more is compensated by moving the pelvis to the oblique position and flexing the knee of the longer leg [8,9]. It results in asymmetric loading of the lower limbs [8,10,11,12]. Effective rehabilitation protocol is needed to change and equalize joint moments and feet loading, both in static and dynamic conditions [13]. In the studies by Heil et al. [9] and Ohlendorf et al. [8] significant differences in postural control were found between the TKR or THR group and healthy controls. The results of the THR group were poorer than those of the TKR group in static conditions [8,9]. Gauchard et al. [11] also reported some postural deficit in balance control during the static test after TKR compared to the control group. They also suggested that knee replacement surgery does not allow accurate orientation of the lower limb and the compensatory role of the knee joint in the regulation of postural control in quiet standing is not restored.

According to Massion [14], postural control depends on several elements. The first is the internal body representation or postural body schema (orientation of body segments and location of the center of mass). The second is multisensory input that regulates the orientation and stabilization of body segments. The third is flexible postural responses or anticipations to recover from a disturbance or postural stabilization during voluntary movement. Small movements accompany the maintenance of any posture. The fact that postural oscillations are small supports the assumption that the system is linear within a limited range of motion [15,16]. While this assumption is correct to some extent, it should be remembered that there is also significant nonlinearity in the postural control system, which tends to be ignored [17].

The most common assessment tool to quantify postural balance is a static standing test with eyes open or closed [1,18]. Center of pressure (CoP) displacements give a possibility to calculate many variables or indicators that can be interpreted as good or poor body balance. Among the most commonly used indicators to assess postural control, some authors [18,19,20] distinguish between those most commonly called linear and those providing indirect insight into the functioning of the nervous system—called nonlinear. Linear tools, such as the CoP path length, sway velocity, and area, quantify the amount of CoP movement during a specific task, independently of their order in the distribution. The nonlinear system approach helps to evaluate different aspects of the CoP data. Nonlinear measures allow for quantifying the regularity and complexity of the system [21,22]. Nonlinear measures include entropy family, fractal dimension, the largest Lyapunov exponent, Hurst exponent, and recurrence quantification analysis (RQA) [18].

Sample entropy (SampEn) is one of the various types of entropy measures. This coefficient is used to determine the regularity of postural sway [23]. The increased values of SampEn indicate a larger irregularity of the CoP, which is more random and less predictable. Lower SampEn values show that the CoP signal is more regular and predictable, which is associated with less complexity of structure [24]. Fractal dimension (FD) is another measure that indicates the complexity of the CoP signal by describing its shape [25]. It shows the complexity and self-similarity of physiological signals [26]. In the case of the CoP trajectory, a change in FD may indicate a change in control strategies for maintaining a quiet stance. The largest Lyapunov exponent (LyE) is a tool characterizing the chaotic behavior of the signal. The human dynamic stability characterized by LyE measures the resistance of the human locomotor control system to perturbations [18]. It quantifies how well an individual can keep a stable posture under perturbations. A higher LyE points to the capability of a more rapid response of balance control in different body movements [27].

Since there are many linear and nonlinear measures that can be used to quantify balance and postural control, it is often problematic to choose some of them that could be sensitive enough to differentiate patients with various medical conditions. Until now, we found no studies comparing postural control between patients after total hip and knee replacement. There are also no studies analyzing which balance and postural control parameters should be used in these groups of patients as a reliable way to differentiate these groups of patients.

The aim of this study was to find the group of linear and/or nonlinear measures that can differentiate healthy people and patients with total knee or hip replacement from each other. This could help to choose the best set of measures that should be calculated from the static balance test to characterize different clinical conditions. It can also suggest which measures are not necessary.

## 2. Materials and Methods

### 2.1. Participants

This study enrolled 49 patients after a total hip replacement (H group) and 53 patients after total knee replacement (K group) due to primary osteoarthritis. All patients were operated on in the Department of Orthopaedics of the Prof. Adam Gruca Independent Public Clinical Hospital in Otwock, Poland. The control group (C group) was 16 healthy persons measured in the Biomechanics Laboratory at the Medical University of Warsaw. None of the measured persons was a professional athlete in the past. The basic data of each group are summarized in Table 1.

The inclusion criteria for the control group included: (1) no balance problems (due to neurological, heart, or musculoskeletal disease), (2) no current musculoskeletal complaints, and (3) written consent to participate in the study. The inclusion criteria for the rest two groups comprised: (1) noncomplicated total knee or hip replacement surgery because of primary osteoarthritis, (2) no other balance problems (due to neurological or heart diseases, vertigo, etc.), (3) no current musculoskeletal complaints other than related to the operated joint, and (4) written consent to participate in the study. All patients from H- and K groups were measured within the first 12 weeks (3–11 weeks) after surgery before the rehabilitation program began.

### 2.2. Ethical Approval

The study protocol was approved by the Bioethics Committee of the Medical University of Warsaw (no. KB/28/2014). The study was conducted according to the ethical guidelines and principles of the Declaration of Helsinki.

### 2.3. Measurement Methods

The postural stability data for each subject were recorded using an AMTI AccuSway (Advanced Mechanical Technology Inc., Watertown, MA, USA) plate with Balance Clinic software. The sample rate was set at 100 Hz. Each person completed three trials of both legs standing with eyes open and three trials of both legs standing with eyes closed. Each trial lasted thirty seconds with a one-minute rest between trials. The results of the patients’ second trials were analyzed. This was performed because patients did not always comply in the first trial and postoperative patients often reported fatigue in the third trial.

### 2.4. Calculation Methods

The study used the linear parameters of CoP path length and three nonlinear measures, sample entropy, fractal dimension, and the largest Lyapunov exponent to assess CoP dynamics. All coefficients were calculated using MatLab software v. R2018b (MathWorks, Natick, MA, USA), separately for mediolateral (ML) and anterior–posterior (AP) CoP data, according to the rules described below. The data for the 30 s trials included 3000 points in each direction.

The 2D CoP path length was calculated in AP and ML directions using the following formulas:

(1) $$\mathrm{CoP}\text{\_}\mathrm{ML}=\sum_{i=2}^{n}\sqrt{{\left(x_{i\;}-x_{i-1}\right)}^{2}}\;\;\mathrm{CoP}\text{\_}\mathrm{AP}=\sum_{i=2}^{n}\sqrt{{\left(y_{i}-y_{i-1}\right)}^{2}}$$

Due to the fact that other commonly used linear parameters (like ellipsis areas, CoP path length) are redundant [28], they were not included in the calculations, as this would distort discrimination analysis and would not give additional information.

SampEn is the negative natural logarithm of the conditional probability that a dataset of length *N*, having repeated itself within a tolerance *r* for *m* points, will also repeat itself for *m* + 1 points, without allowing self-matches:

(2) $$SampEn\left(m,r,N\right)=-ln\left(\frac{A^{m}\left(r\right)}{B^{m}\left(r\right)}\right)$$

*B* represents the total number of matches of length m while *A* represents the subset of *B* that also matches for *m* + 1. For calculating the SampEn, MatLab codes obtained from the Physionet tool [29] were used, with “default” parameter values: *m* = 2 and *r* = 0.2 × (standard deviation of the data).

FD was calculated using Higuchi’s algorithm [30]. Higher FD values are associated with the greater complexity of a time series.

LyE was calculated to detect chaotic system dynamics, using the following equation:

(3) $$d\left(t\right)=Ce^{LyEt}\;$$

In LyE equation *d*(*t*) is the average divergence at time t and *C* is a constant that normalizes the initial separation [31]. A positive LyE value is often considered a necessary condition for the presence of chaos in a given system. If LyE is zero, it means the system is conservative, (i.e., there is no dissipation). If the system is dissipative, the LyE value is negative.

### 2.5. Statistical Analysis

Statistical analysis was performed using Statistica v. 13.1 (TIBCO Software, Inc., Palo Alto, CA, USA), PQStat 2021 software v. 1.8.2.238 (PQStat Software, Poznań, Poland), and GRETL software v. 2019a (Free Software Foundation, Boston, MA, USA). The threshold for statistical significance was assumed at *p* < 0.05.

The Shapiro–Wilk test was used to assess the normality of all data distributions. Next, it was checked if variance matrices of variables are homogeneous across groups. For inter group comparison, one-way ANOVA with Tukey’s post hoc test was used.

Tree cluster analysis was used for grouping patients according to analyzed linear and nonlinear measures from both tests (EO, EC). The grouping was performed using connectivity-based clustering with a weighted group method with medians with Euclidean distance. There were no assumptions about the number of groups.

Next, discriminant analysis was used to determine which linear and nonlinear parameters discriminate between three groups (K, H, and C). Sixteen variables were included in the analysis: SampEn, FD, LyE, and CoP path from eyes open and eyes closed tests, each calculated separately for AP and ML directions. The forward stepwise analysis was used to build the discriminant model.

In the end, regression analysis with a method of least squares was performed for each balance variable to define what each balance parameter is dependent on. The best model was chosen upon the Akaike information criterion.

## 3. Results

### 3.1. Cluster Analysis

**Figure 1:**
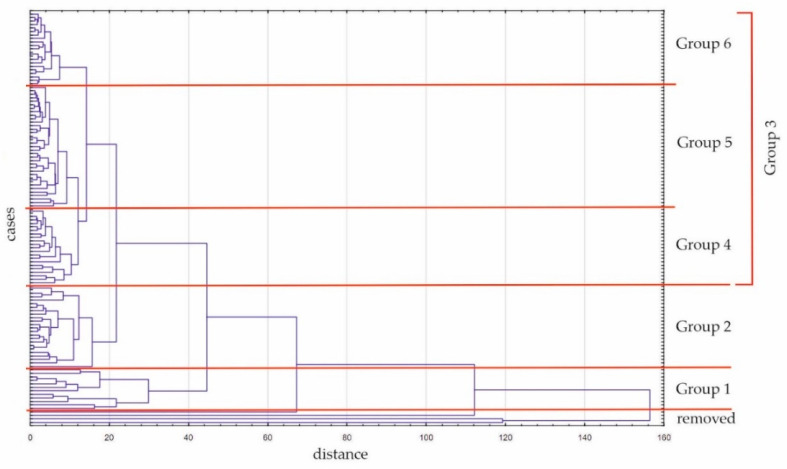
The tree graph from cluster analysis. The *x*-axis presents distance, while the *y*-axis includes participants of the study.

Initially, four participants were removed for further analysis, because they did not connect closely with other participants. Then, three groups were extracted (groups no. 1, 2, and 3). None of these groups corresponded to the clinical group (H, K, or C). Patients after hip replacement were classified into groups 2 and 3, patients after knee replacement into groups 1, 2 or 3 (one patient was removed), while controls were included in group no. 3 only (three persons were removed). Then, group no. 3 was divided into three groups according to the tree graph (Figure 1) to analyze the data in detail. Table 2 shows the allocation of participants into five groups.

Due to the fact that no person from the C group was allocated to the group no. 1 or 2, we checked the differences between groups no. 1, 2, and 3. No significant differences were found between those groups other than balance parameters.

### 3.2. Discriminant Analysis

The best model was reached in step eight and included eight variables (Table 3). Overall, the discrimination between three groups (H, K and C) was highly significant (Wilks’ Lambda: 0.0354; F (16,216) = 58.247; *p* < 0.0001). The percentage of correctly classified cases is presented in Table 4. Classification functions are presented in Table 5. Model variables are summarized according to test (EO, EC) and direction (ML, AP) in Table 6.

### 3.3. Regression Analysis

Analysis of the regression for nonlinear measures showed that sample entropy depends on anthropometrical variables (age, *p* = 0.027 and BMI, *p* = 0.021), test condition (EO/EC, *p* = 0.005), as well as group (H/K/C; *p* < 0.001). Fractal dimension depended only on the group (H/K/C; *p* < 0.001) and gender (*p* = 0.043). The largest Lyapunov exponent depended on gender (*p* = 0.045), BMI (*p* = 0.020), index direction (AP/ML, *p* < 0.001) and group (H/K/C; *p* < 0.001). COP path length depended on belonging to the H group (*p* = 0.001) and the direction (AP/ML; *p* < 0.001). None of the analyzed variables depended on time after surgery.

## 4. Discussion

The aim of this study was to find the group of linear and/or nonlinear measures that can differentiate healthy people and patients with total knee or hip replacement from each other. Three types of statistical analyses were performed to achieve this goal. Cluster analysis did not result in correct allocation to the groups according to all variables that were calculated from the balance test with eyes open and closed, although all controls were classified into one group in the three-groups model. However, this group also contained patients after THR and TKR. The result of the discriminant analysis was an eight-variables model including the largest Lyapunov exponent (ML EO, ML EC, and AP EC), fractal dimension (AP EO, ML EC, AP EC), CoP path AP EC and SampEn AP EC. The model was correct in 76.3% of cases. Regression analysis showed that all nonlinear variables depend on the group, while CoP path length is different only in the H group. Some influence of anthropometric parameters (gender, BMI, age) as well as direction (AP or ML) was also indicated.

Differences in postural control in patients after total knee or hip replacement and healthy controls were confirmed by Heil et al. [9] and Ohlendorf et al. [8]. In both studies, the CoP path from static measurement was significantly longer in the study group than in the controls. These studies were made by the same research group as well as the same protocol, and therefore, it can be easily seen that patients after TKR reached better results than those after THR. In our study, we did not analyze which group was better, but significant differences can be confirmed by the results of regression analysis. CoP path length was significantly dependent on belonging to the H group, which suggests that results in this group were different from those achieved by participants after TKR or healthy controls.

To the best of our knowledge, no publications are analyzing the ability of a group of linear and/or nonlinear balance measures to different groups with various clinical conditions. However, there are some scientific reports analyzing the usefulness of different variables in discriminating different groups of patients, mostly fallers from non-fallers or older from young adults.

Many publications suggest that nonlinear measures can measure the amount of attention paid to maintaining balance in certain conditions [18,22,32,33]. Introducing mainly nonlinear measures from the eyes-closed test to the discrimination model suggests that this test is more reliable and sensitive than the test with eyes open. For most people standing without visual feedback is a more demanding task and therefore requires more attention, which should decrease the values of nonlinear measures, especially sample entropy [18,32]. This can suggest that standing with eyes closed is a better way to assess the amount of regularity of CoP movement and attention paid to maintain balance.

The largest Lyapunov exponent shows the ability to adapt to the environment by investigating how the musculoskeletal system states change over time in terms of exponential divergence/convergence of initially nearby trajectories [18]. Our results clearly show that this variable has a large impact on the discrimination model, both when calculated from the EO test (LyE ML) and EC test (LyE ML, LyE AP). Additionally, regression analysis confirmed the high dependence of LyE values on the group. This suggests that differences between three clinical groups (THR, TKR, and healthy controls) comprise differences in the ability to adapt to the environment. Higher LyE values suggest a better (faster) response of balance control in different body movements [27]. Significant differences in LyE values between different study groups were confirmed by Ghofrani et al. [34], Huisinga et al. [35], and Liu et al. [36].

Fractal dimension calculates the complexity and irregularity of the signal over time and its values can be interpreted as an ability to synergistically modulate three systems involved in maintaining posture—the somatosensory, visual, and vestibular systems. Kędziorek and Błażkiewicz [18] suggest that the fractal dimension is not sensitive enough to detect an age group difference. Results of the discriminant analysis showed that FD can be useful in determining group classification, while calculated from the EC test. Regression analysis also confirmed that this variable is group dependent. FD also depended on gender, but the direction (AP/ML) did not influence the result. The latter fact was confirmed by Szafraniec et al. [37] by comparing results of FD in AP and ML directions.

Montesinos et al. [38] showed that sample entropy can discriminate fallers from non-fallers and younger from older adults for AP direction and a specific combination of calculating parameters (*m*, *r*) only. Borg and Laxaback [39] also found significant differences between young and older adults in SampEn AP. Regression analysis in our study showed that SampEn depends, among others, on age. Raffalt et al. [40] found a group (ankle instability/controls) significant effect on sample entropy values. This can be partially confirmed in our study, where sample entropy for AP direction from the EC test was included in the discriminant function, although it was not statistically significant. In the studies of Szafraniec et al. [37] and Raffalt et al. [40] the influence of direction (AP/ML) on SampEn values was demonstrated. In our study, regression analysis did not confirm this, but on the other hand, only SampEn AP values were included in the discrimination model.

Linear measures are more often used to assess balance in clinical practice, than nonlinear measures. Borg and Laxaback [39] found out that CoP ML amplitude can discriminate between elderly fallers and non-fallers, but only for foam and head extension conditions. Other differences (between young and older adults) were not significant. Our study was performed only in static conditions and the influence of CoP path in AP direction in EC-test in discriminating patients after THR, TKR, and healthy controls were confirmed, although it should be pointed out that regression analysis showed only the H group influence on CoP path values.

Analysis of classification functions clearly shows that coefficients for healthy controls are significantly different from those for H and K groups. Additionally, the percentage of correctly classified participants from the C group is 100%, which confirms that this group reached completely different results from those of patients after joint replacement, and therefore, it was easier to build the discrimination model that correctly classified healthy controls. Differentiation between THR and TKR groups is less effective, reaching above 70% of correctly classified cases and coefficient values show that these two groups are more similar to each other.

Cluster analysis showed that some patients from THR and TKR groups are similar regarding all analyzed variables together to healthy controls and these were classified together to group no. 3. However, some of them (eight patients from the H group and twenty-eight patients from the K group) were classified into other two groups that included no healthy controls. Probably, there are other clinical, anthropometrical, or psychological factors that were not analyzed in this study and which influence the results of balance tests.

Some limitations of this study have to be acknowledged. First of all, significant differences in age between the three groups could have contributed to worse classifications of the groups. However, this was not confirmed in post hoc calculations. Secondly, the analyzed groups differ also in BMI, but this is hard to avoid, as obese and overweight people are more likely to have knee or hip osteoarthritis [4]. Thirdly, it seems to be clear that there are some other factors that can influence the classification of the groups that were not included in this study. Probably, including the results of physical examination, additional measurements such as body composition or densitometric tests, clinical assessment scales, or gait analysis in future studies are needed. It would be also worthwhile to analyze the medical history of the patients in a more detailed way.

## 5. Conclusions

Inclusion of the variables calculated from the standing with eyes closed test into the discrimination model suggests that standing with eyes closed is a better way to assess the amount of regularity of CoP movement and attention paid to maintain balance. The obtained results also suggest that nonlinear measures better differentiate TKR and THR patients from healthy controls than linear variables and therefore, it is worthwhile to include nonlinear measures in patient balance analysis, especially the largest Lyapunov exponent and fractal dimension. This study did not conclude with a clear result and the set of parameters found in discriminant analysis is probably not the best one, although it can easily differentiate healthy controls of patients after joint replacement in the lower limb. In further studies, it is recommended to include the results of physical examination, clinical assessment scales, or gait analysis for more satisfying results.

## Figures and Tables

**Table 1:** Mean ± standard deviation of anthropometric data of H-, K- and C groups.

	H Group (N = 49)	K Group (N = 53)	C Group (N = 19)
Gender	28 women, 21 men	34 women, 19 men	15 women, 1 man
Age (years)	63.7 ± 8.8 * ^HKC^	68.4 ± 6.3 * ^HKC^	53.0 ± 7.6 * ^HKC^
Bodyweight (kg)	81.5 ± 16.0	85.7 ± 16.1 * ^KC^	74.8 ± 16.3 * ^KC^
Height (cm)	167.5 ± 10.1	166.1 ± 11.5	164.9 ± 4.9
Body Mass Index (kg/m^2^)	28.8 ± 4.2 * ^HK^	30.9 ± 3.9 * ^HK,KC^	27.4 ± 5.3 * ^KC^

* *p* < 0.01 in ANOVA test with post hoc Tukey’s test; letter (H, K or C) indicates group in Tukey’s test.

**Table 2:** Numbers of participants from H, K, and C groups allocated to five groups created based on cluster analysis.

	Group 1	Group 2	Group 3		
			Group 4	Group 5	Group 6
**H group**	0	8	13	20	8
**K group**	12	16	4	14	6
**C group**	0	0	5	1	7

**Table 3:** Results of the discriminant analysis—model with 8 variables. Non-significant variables are marked with italic.

Variable	Wilks’ Lambda	F to Remove (2.108)	*p*-Value
LyE ML EO	0.042	10.355	< 0.0001
FD AP EO	0.039	5.671	0.0045
CoP path_ML EC	0.045	14.642	< 0.0001
LyE ML EC	0.040	7.254	0.0011
FD ML EC	0.038	3.492	0.0339
LyE AP EC	0.041	8.824	0.0003
*FD AP EC*	*0.037*	*1.693*	*0.1889*
*SampEn AP EC*	*0.037*	*2.795*	*0.0656*

LyE—the largest Lyapunov exponent, FD—fractal dimension, SampEn—sample entropy, EO—test with eyes open, EC—test with eyes closed.

**Table 4:** Results of the discriminant analysis—percentage and number of correctly classified participants.

	% Correctly Classified	H Group (N)	K Group (N)	C Group (N)
**H group**	73.5	36	13	0
**K group**	71.7	15	38	0
**C group**	100	0	0	16
**together**	76.3	51	51	16

**Table 5:** Results of the discriminant analysis—classification functions for each group.

Variable	H Group *p* = 0.4153	K Group *p* = 0.4492	C Group *p* = 0.1356
LyE ML EO	53.417	57.047	127.685
FD AP EO	150.088	142.413	102.269
CoP path_ML EC	−0.767	−0.770	−1.746
LyE ML EC	37.833	38.242	99.582
FD ML EC	98.003	98.237	129.198
LyE AP EC	−0.788	3.063	44.772
FD AP EC	257.086	263.192	249.053
SampEn AP EC	−225.615	−220.608	−177.377
const.	−348.296	−348.863	−413.511

LyE—the largest Lyapunov exponent, FD—fractal dimension, SampEn—sample entropy, EO—test with eyes open, EC—test with eyes closed.

**Table 6:** Discriminant model summarized according to test conditions and direction. Non-significant variables are marked with italic.

	ML	AP
**Test with eyes open (EO)**	LyE	FD
**Test with eyes closed (EC)**	LyE, FD	CoP path, LyE, *FD*, *SampEn*

LyE—the largest Lyapunov exponent, FD—fractal dimension, SampEn—sample entropy.

## Data Availability

The measurement data used to support the findings of this study are available from the corresponding author upon request.
